# Panton-Valentine Leukocidin–Positive *Staphylococcus aureus* in Family and Pet Cat 

**DOI:** 10.3201/eid3008.231255

**Published:** 2024-08

**Authors:** Astrid Bethe, Anne-Kathrin Schink, Julian Brombach, Lennard Epping, Torsten Semmler, Susanne Reinhardt, Ernst Molitor, Svenja Müller, Julian Balks, Robin Köck, Stefan Schwarz, Birgit Walther, Antina Lübke-Becker

**Affiliations:** Freie Universität Berlin, Berlin, Germany (A. Bethe, A.-K. Schink, J. Brombach, S. Schwarz, A. Lübke-Becker);; German Environment Agency, Berlin (A. Bethe, B. Walther);; Laboratory Diagnostics Germany, Cuxhaven, Germany (A.-K. Schink);; Robert Koch Institute, Berlin (L. Epping, T. Semmler, B. Walther);; Kleintierpraxis am Kenntemichplatz, Troisdorf, Germany (S. Reinhardt);; University Hospital, Bonn, Germany (E. Molitor, S. Müller, J. Balks);; Universitätsmedizin, Essen, Germany (R. Köck)

**Keywords:** Staphylococcus aureus, Panton-Valentine leukocidin, PVL animals, cat, zoonoses, antimicrobial resistance, bacteria, staphylococci, Germany

## Abstract

Continued detection of Panton-Valentine leukocidin–positive *Staphylococcus aureus* in samples from a family with severe repeated skin infections and their pet cat suggests transmission between the family and the cat. Decolonizing the pet led to successful elimination of the bacteria from the household. Clinicians should consider pet cats as possible reinfection sources.

Panton-Valentine leucocidin (PVL)–producing *Staphylococcus aureus* (PVL-SA) is typically associated with skin and soft tissue infections, such as abscesses (*1*). Transmission occurs between persons in close contact, often causing clusters of community-onset infections, especially within families (*1*). PVL-SA contains the genes *lukS* and *lukF*, which encode 2 toxin components capable of forming a pore-like octamer on neutrophil membranes, leading to cell lysis, local inflammation, and tissue damage (*2*). Because staphylococci colonize the nares, pharynx, and skin, infection reemergence occurs in the case of abrasions or wounds, enabling PVL-SA to cross the skin barrier. Topical decolonization of the skin and other body sites during or after acute PVL-SA infections is part of the therapy (*3*). In this article, we describe the successful decolonization of a family in Germany, comprising 2 adults and 2 children, who experienced severe recurrent PVL-SA infections and multiple unsuccessful decolonization attempts with increased hygiene measurements over an extended period. 

The consultant laboratory for methicillin-resistant staphylococci in veterinary practice and clinic (CL-MRS-VPC), located at the institute of microbiology and epizootics, Freie Universität Berlin, was contacted in January 2022 because a family affected by severe repeated soft tissue infections caused by PVL-SA had undergone 3 unsuccessful decolonization attempts. Everyone in the family had experienced multiple (5–15/person) skin abscesses caused by PVL-SA since 2017. The 3 unsuccessful ambulatory decolonization attempts of the family were performed by the University Hospital in Bonn, Germany, according to a standard protocol (Charité, https://hygiene.charite.de/forschung/arbeitsgruppen/ag_pvl_bildender_staphylococcus_aureus). A review and assessment of possible reinfection sources by the clinicians led to the initial sampling of 2 household cats. Samples from both cats (oral, nasal, inguinal, perianal, and rectal) were screened at CL-MRS-VPC.

We cultured all swab samples as previously described (*4*). We identified the *S. aureus* isolates by using matrix-assisted laser desorption/ionization time-of-flight mass spectrometry (Bruker, https://www.bruker.com). We conducted antimicrobial susceptibility testing by using the VITEK2 system (bioMérieux, https://www.biomerieux.com) and Clinical and Laboratory Standards Institute clinical breakpoints (*5*). We used PCR on the *S. aureus* isolates recovered to detect PVL-encoding genes (*lukF* and *lukS*) (*6*). We isolated methicillin-susceptible *S. aureus* (MSSA) from the oral cavity and nostrils of both cats. Cat 1 was confirmed as a carrier of PVL-positive MSSA, and cat 2 carried PVL-negative MSSA (Table; Figure). We further investigated whether the feline MSSA strains were circulating in the family by conducting whole-genome sequencing of both feline strains and PVL-positive and PVL-negative MSSA strains obtained from samples of the human family members (Table).

**Table:** Antimicrobial susceptibility testing results of *Staphylococcus aureus* isolates recovered from a family who had repeated soft-tissue infections caused by Panton-Valentine leukocidin–producing *S. aureus* and their pet cats*

Isolate	IMT51844	IMT51843	IMT51535	IMT51669	IMT51533	IMT51534	IMT51668	IMT51681
Source	Child	Parent	Cat 2	Cat 2	Cat 1	Cat 1	Cat 1	Cat 1
Sample site	Nose	Inguinal	Mouth	Nose	Mouth	Nose	Nose	Nose
Date	2021 Nov	2021 Nov	2022 Mar	2022 Mar	2022 Mar	2022 Mar	2022 Mar	2022 Mar
ST	45	8	45	45	8	8	8	8
PVL	–	+	–	–	+	+	+	+
Antimicrobial drug MIC, µg/mL								
CIP	0.12	16	0.12	0.25	16	16	16	16
ENR	0.12	4	0.12	0.12	4	4	4	4
MAR	0.25	16	0.25	0.25	16	16	16	16
GEN	1	0.25	0.5	1	0.25	0.25	1	0.25
SXT	0.06/1.19	0.06/1.19	0.06/1.19	0.06/1.19	0.06/1.19	0.06/1.19	0.06/1.19	0.06/1.19
TET	0.25	32	0.5	1	64	32	32	64
DOX	0.5	4	0.5	0.5	4	4	4	4
PEN	0.03	0.03	0.03	0.03	0.03	0.06	0.06	0.06
AMP	0.12	0.12	0.25	0.12	0.12	0.12	0.25	0.25
AMC	0.25/0.12	0.25/0.12	0.25/0.12	0.25/0.12	0.25/0.12	0.25/0.12	0.25/0.12	0.25/0.12
OXA	0.12	0.25	0.12	0.25	0.25	0.25	0.5	0.25
ERY	1	>64	0.5	0.5	>64	32	>64	>64
CLI	0.25	0.25	0.25	0.25	0.12	0.12	0.25	0.12

*AMC, amoxicillin/clavulanate; AMP, ampicillin; CIP, ciprofloxacin; CLI, clindamycin; DOX, doxycycline; ENR, enrofloxacin; ERY, erythromycin; GEN, gentamicin; MAR, marbofloxacin; OXA, oxacillin; PEN, penicillin; PVL, Panton-Valentine leukocidin; ST, sequence type; SXT, sulfamethoxazole/trimethoprim; TET, tetracycline; +, positive; –, negative.

We performed a de novo assembly of the paired-end reads and annotated the resulting genomes as previously described (*7*). We generated an alignment of the maximum common genome from the study set to identify single-nucleotide polymorphisms (SNPs). The SNP analyses showed close clonal relationships of both the PVL-MSSA, assigned to sequence type 8 (range 10–12 SNPs), and the PVL-negative MSSA strains, assigned to sequence type 45 (range 1–7 SNPs), isolated from human and feline samples. The *lukS* and *lukF* genes of the PVL-MSSA isolate shared 100% nucleotide sequence identity and coverage with the corresponding genes in strain USA300 FPR3757 (GenBank accession no. 87125858), an epidemic clone of community-acquired methicillin-resistant *S. aureus* (*8*).

According to regulations in Germany for research with animal subjects, attempts to decolonize cats by using amoxicillin/clavulanate do not need approval (Landesamt für Gesundheit und Soziales, Berlin, pers. comm., letter, 2023 Feb 6). We performed an initial attempt to decolonize the cats on the basis of antimicrobial susceptibility testing results involving oral administration of amoxicillin/clavulanate for 10 days (Figure). The decolonization attempt was accompanied by hand washing and isolation of the cats indoors.

**Figure:**
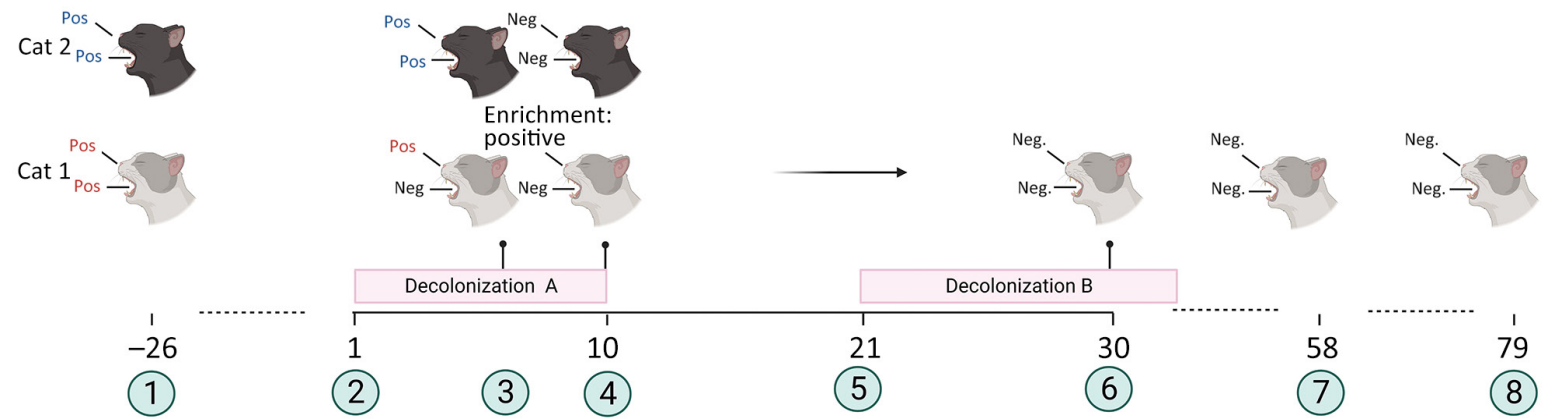
Timeline and overview of a successful decolonization attempt of 2 household cats colonized with methicillin-susceptible *Staphylococcus aureus*, Germany. The family suffered from repeated soft-tissue infections caused by PVL-SA. Cat 1 was colonized with PVL-SA; cat 2 was colonized with PVL-negative SA. Decolonization period A consisted of oral administration of amoxicillin/clavulanic acid for 10 days. Decolonization period B consisted of parenteral administration of amoxicillin for 14 days. 1, initial screening for SA; 2, start of decolonization period A; 3, screening results at day 7 of decolonization period A; 4, screening results at day 10 of decolonization period A; 5, start of decolonization period B; 6, screening result of cat 1 during decolonization period B; 7, screening results on day 58 from the start of decolonization period A; 8, screening results on day 79 from the start of decolonization period A. Red text indicates positive for PVL-SA; blue text indicates positive for PVL-negative *S. aureus*. Figure created with Biorender (https://www.biorender.com; license BW 27.06.2023). Enrichment: Neg, negative for *S. aureus*; Pos, positive; Positive, positive for PVL-SA after enrichment step in liquid medium; PVL, Panton-Valentine leukocidin; PVL-SA, PVL-positive *S. aureus*.

Samples from cat 2 were negative for *S. aureus* after 10 days, but cat 1, which was PVL-SA–positive, required a second course of antimicrobial drugs (Figure). Because cat 1 refused oral administration of antimicrobial drugs for the second decolonization, a 14-day course of amoxicillin was administered parenterally. Swab samples from cat 1 taken on days 58 and 79 after the second decolonization yielded negative results (Figure). A fourth attempt to decolonize the entire family was conducted in parallel and was successful, according to samples taken from the family 1 week, 3 months, and 6 months after decolonization.

When recurrent PVL-SA–associated soft tissue infections occur within a family and increased hygiene measures and decolonization attempts are repeatedly unsuccessful, clinicians should consider that pets may be involved in the transmission circle. Because the close phylogenetic relationship of the human and feline isolates in this case strongly suggested transmission, our findings highlight the importance of considering colonized or infected pets as a potential source of reinfection for humans during *S. aureus* decolonization attempts, as previously reported (*9*,*10*). In such cases, cat decolonization attempts require antimicrobial drugs that are well tolerated and approved as first-line treatments in veterinary medicine. Feasibility of treatment and animal welfare of the feline outpatients should be taken into consideration.

## References

[1] Von Dach E, Diene SM, Fankhauser C, Schrenzel J, Harbarth S, François P. Comparative genomics of community-associated methicillin-resistant *Staphylococcus aureus* shows the emergence of clone ST8-USA300 in Geneva, Switzerland. J Infect Dis. 2016;213:1370–9. 10.1093/infdis/jiv489 26464204

[2] Shallcross LJ, Fragaszy E, Johnson AM, Hayward AC. The role of the Panton-Valentine leucocidin toxin in staphylococcal disease: a systematic review and meta-analysis. Lancet Infect Dis. 2013;13:43–54. 10.1016/S1473-3099(12)70238-4 23103172 PMC3530297

[3] Leistner R, Hanitsch LG, Krüger R, Lindner AK, Stegemann MS, Nurjadi D. Skin infections due to Panton-Valentine leukocidin–producing *S. aureus.* Dtsch Arztebl Int. 2022;119:775–84. 36097397 10.3238/arztebl.m2022.0308 PMC9884843

[4] Hanselman BA, Kruth SA, Rousseau J, Weese JS. Methicillin-resistant *Staphylococcus aureus* colonization in schoolteachers in Ontario. Can J Infect Dis Med Microbiol. 2008;19:405–8. 10.1155/2008/284239 19436569 PMC2663470

[5] Clinical and Laboratory Standards Institute. Performance standards for antimicrobial disk and dilution susceptibility tests for bacteria isolated from animals. 5th ed. CLSI supplement VET01S. Wayne (PA): The Institute; 2020.

[6] Lina G, Piémont Y, Godail-Gamot F, Bes M, Peter MO, Gauduchon V, et al. Involvement of Panton-Valentine leukocidin-producing *Staphylococcus aureus* in primary skin infections and pneumonia. Clin Infect Dis. 1999;29:1128–32. 10.1086/313461 10524952

[7] Huber C, Stamm I, Ziebuhr W, Marincola G, Bischoff M, Strommenger B, et al. Silence as a way of niche adaptation: *mec*C-MRSA with variations in the accessory gene regulator (*agr*) functionality express kaleidoscopic phenotypes. Sci Rep. 2020;10:14787. 10.1038/s41598-020-71640-4 32901059 PMC7479134

[8] Diep BA, Gill SR, Chang RF, Phan TH, Chen JH, Davidson MG, et al. Complete genome sequence of USA300, an epidemic clone of community-acquired meticillin-resistant Staphylococcus aureus. Lancet. 2006;367:731–9. 10.1016/S0140-6736(06)68231-7 16517273

[9] Vincze S, Brandenburg AG, Espelage W, Stamm I, Wieler LH, Kopp PA, et al. Risk factors for MRSA infection in companion animals: results from a case-control study within Germany. Int J Med Microbiol. 2014;304:787–93. 10.1016/j.ijmm.2014.07.007 25130703

[10] Sing A, Tuschak C, Hörmansdorfer S. Methicillin-resistant *Staphylococcus aureus* in a family and its pet cat. N Engl J Med. 2008;358:1200–1. 10.1056/NEJMc0706805 18337614

